# Narrative inquiry of translators’ identities: A study of meaning-making in narrating knowledge

**DOI:** 10.3389/fpsyg.2023.1070178

**Published:** 2023-01-30

**Authors:** Qiang Geng

**Affiliations:** Institute of Corpus Studies and Applications, Shanghai International Studies University, Shanghai, China

**Keywords:** narrative inquiry, human-centeredness, translation experience, narrative knowledge, metanarrative, professional narrative, personal narrative, narrative identities

## Abstract

Translators have generated retrospective accounts of their working experience, which contribute to an expansive corpus of knowledge on translation. A plethora of research has explored how this knowledge could enrich our perception of varied questions concerning translation process, strategies, norms, and other social and political respects within conflictual settings in which translation has engaged. In contrast, few attempts have been made to gain a translator-centered understanding of what this knowledge could mean for its narrators. In line with narrative inquiry, this article proposes a human-centered approach to translator’s knowledge narrating and a shift from positivistic to post-positivistic investigation into specific questions about how translators make sense of who they are as well as the meaning of their lives by structuring their experiences into a sequential and meaningful narrative. The general question is what strategies are employed to construct what types of identities. A holistic and structured analysis of five narratives by senior Chinese translators involves macro and micro dimensions. With a view to methods employed by scholars in different fields, the study identifies four types of narratives, namely, personal, public, conceptual/disciplinary, and metanarrative, which are used throughout our cases. Micro-analysis of narrative structure demonstrates that life events are often arranged in a chronological sequence, among which critical events are favored to indicate a turning point or crisis for transformation. Storytellers tend to adopt strategies of personalizing, exemplifying, polarizing, and evaluating to construct their identities and what translation experience means to them. This article concludes that apart from communicating translation knowledge, translators make sense of what translation experience means to them as a professional translator and more importantly as a real person living through social–cultural–political vicissitudes, thus contributing to a more translator-centered vision of translation knowledge.

## 1. Introduction

Translators’ retrospective musings about their own translation experience have historically been a major source of translation expertise (D’hulst and Gambier, 2018). Continued efforts have been made to collect paratextual and metatextual documents from which such knowledge is mainly formed (Luo, 1984; Lefevere, 1992; Chen, 2000; Robinson, 2002; Chan, 2004; Wang, 2004; Baer and Olshanskaya, 2014). Such expertise is valued highly for shedding light on translation processes, methods, and norms (Lefevere, 1992, p. 109; Bassnett, 1998). In contrast, the extrinsic approach emphasizes contextuality in knowledge production and how translation knowledge has mediated through conflictual circumstances (Cheung, 2003).

In addition, current studies also tend to interpret the value of such knowledge against translation studies as a discipline. However, in the 1980s when it became a success, an epistemological crisis appeared in translation knowledge legitimization. The traditional philological approach to translation has undergone a paradigmatic shift to more scientific research, which indicates that translation knowledge is supposed to be objective, systematic, and quantitative. Against this modernistic quest for scientific knowledge, the translator’s account of their own translation experience has been subjected to a searing critique due to its impressionistic and anecdotal features (Dong, 1951; Guo, 1987; Wong, 1999; Chan, 2004). As a result, such conventional knowledge had to face a growing amount of suspicion about its legitimacy as a form of professional knowledge and thereby unmistakably withdrew into disciplinary peripherality.

Apparently, within a discipline with a positivistic tendency, translators’ working experience narrative is interpreted at best as a “defective” type of knowing. However, this tunneled vision restrains our divergent perceptions of the heterogeneous nature of translation knowledge, which will be articulated in this article. In addition, a simplistic vision also paralyzes a rethinking of how “spin-off” data like translators’ self-narratives of their working experience could be critically bound up with more significant questions about translators’ identities, a topic recently gaining much momentum and complexity in a globalized world with high mobility among people, things, and ideas (Venuti, 2005; Cronin, 2006; André, 2020; Almanna and Gu, 2021; Heino, 2021). In addition, some researchers view translators as social actors in their specific localities, focusing on the interaction between translators’ translations and their multifaceted roles in various modern institutions (Snell-Hornby, 1999; Voinova and Shlesinger, 2013; Yu, 2019). In these studies, however, translators’ experience narrative has been invariably underinvestigated, if not ignored. Therefore, this negligence necessitates a dynamic view to uncover new grounds for narrative knowledge within translation studies and beyond.

The above literature review demonstrates possible gaps this article tries to fill. First and foremost, translators’ experience narrative has been primarily investigated within translation studies with a focus on the epistemological values such knowledge may have in enriching our perceptions of translation products, processes, and functions. However, this tendency to highlight events, text, and context fails to put humans as the center of knowledge production, running against the rising interest in translator studies articulated by Chesterman (2009) and Kaindl et al. (2021). On the other hand, in today’s modernistic and scientific regime of knowledge production within translation studies, the knowledge based on translators’ experience narrative has often been undervalued as a deviation from modern knowledge due to its features of being impressionistic and anecdotal. As a result, the narrative aspects involving knowledge production are less fully discussed in narrative approaches to translation studies. A wide range of questions pertaining to life stories and how those stories are deployed for constructing identities and making meaning of life are, thus, less queried.

Drawing on framework and methods from translation studies, and in particular narrative inquiry, we would highlight the importance of human-centeredness in our perception of the nature of translators’ self-narrative of their own translation experience. In respect of this assumption, we tend to argue first and foremost that translators’ translation experience narrative is, in one way or another, a form of narrative generated in specific temporal-spatial dimensions for a wide range of readers. It is best to be viewed as narrative knowledge, by which empirical knowledge is conveyed *via* accounting of lived experience by storytellers. It is not entirely congruent with the modernized knowledge industry typically featured with thesis, papers, and/or projects.

Nevertheless, both types of knowledge complement each other and contribute to the whole gamut of translation knowledge. Analysis of five specific cases selected from Chinese contexts in this article reveals that translators’ knowledge narrative often mixes, among other things, know-how techniques with a personal account of life stories. Briefly, as a hybrid text, its purpose is not simply to communicate knowledge on how to translate but also to construct who they are and make meaning of their life as a social agent *via* storytelling. Indeed, narrative knowledge is not perfect compared to more scientific and logical genres of professional knowledge. Using a holistic method to study what narrative is about, this article attempts to investigate the nature, structure, and function of translators’ knowledge narratives and tries to ask specific questions concerning what strategies are adopted to construct what kinds of identities. In putting the narratives back into the contexts where they were generated and considering translators’ accounts referring to events and figures in history, this article will articulate what knowledge narratives mean to their narrators who have created their life’s meaning by telling stories about what they did, how they feel, and what they hope for. By foregrounding a translator-centered vision of knowledge narrative, this article will contribute to a more humanistic conceptualization of translation knowledge and translation history.

## 2. Theoretical framework and methods

This study first uses narrative inquiry as a theoretical underpinning. As a field of study, narrative inquiry is best viewed as a family of cross-disciplinary enterprises, with divergent epistemological propositions, questions, and methods (Chase, 2005, p. 651; Webster and Mertova, 2007, p. 19; Wells, 2011, p. 7; De Fina and Georgakopoulou, 2012, p. 5). As a denial of positivism domineering in the studies of human sciences in the 1980s, it triggers a humanistic approach to narrative (Fisher, 1985, p. 3; Plummer, 2001, p. 198; Pinnegar and Daynes, 2007, p. 348; Andrews et al., 2008, p. 449; Hyvärinen, 2008, p. 9; Bochner and Riggs, 2014), and thus views the experience of individuals or groups as narrative or story. It is due to this human-centeredness that we tend to articulate translators’ recounting of their translation experience as a form of narrative and further consider what it could mean to translators as storytellers.

The concept of narrative has no unified definition to cover all applications (Czarniawska, 2004; Riessman, 2008). Within psychological research, it is often viewed as a cognitive scheme or organizing principle by which human beings arrange their life in a meaningful way (Sarbin, 1986; Polkinghorne, 1988).

This diversity involving the definition of narrative also affects how we categorize it into different types. Next to Bruner’s (1986) bi-typology of knowledge into the narrative and paradigmatic, we have many alternative schemes. Somer and Gibson’s, (1994 p. 60–63) categorical analysis of four dimensions of narrativity, namely, ontological, public, conceptual, and “meta” narrativity, are further modified by Baker (2006) in her narrative-based approach to translation. Ontological narratives, or narratives of the self, are viewed as “personal stories we tell ourselves about our place in the world and our history. These stories both constitute and make sense of our lives” (Baker, 2006, p. 28). Public narratives, as Baker (2006, p. 33) illustrates, refer to “stories elaborated by and circulating among social and institutional formations larger than the individual, such as the family, religious or educational institution, the media and the nation.”

However, Somer and Gibson’s list expels literature as “one of the most powerful institutions for disseminating public narratives in any society” (Baker, 2006). Baker (2006, p. 39) describes the conceptual or disciplinary narrative as “the stories and explanations that scholars in any field elaborate for themselves and others about their object of inquiry.” Finally, meta-(master) narrative briefly involves narratives that gain wider circulation and have significant impacts on people and society (Baker, 2006, p. 45). The boundaries between these types sometimes are flexible because, for example, the public narrative can evolve into a metanarrative if it has gained dominance in society due to social-political factors.

The narrative often fulfills a plethora of functions, one of which is to construct identities individually or collectively. A nation as an imagined community (Anderson, 2006) normally appropriates stories and tales to forge a sense of belongings among its people. Those narratives can take multivarious formations in history books, anthems, film episodes, and touchable memorials. During the process, several strategies are often utilized. As Schubert (2010) illustrates in a study of political speech, storytellers would normally adopt personalizing, integrating, exemplifying, and polarizing in their narratives. In personalizing, storytellers focus on their “personal anecdotes or autobiographical flashbacks” (Schubert, 2010, p. 147) as a way of establishing solidarity with the audience. Integrating is to highlight what a nation or party has achieved for public goodness.

Furthermore, with exemplifying, narrators would mention a single individual as an instance of the point speakers intend to make (Schubert, 2010, p. 150). Finally, political speakers tend to use polarizing to draw a clear line between us and political opponents so future actions can be taken toward them. Apparently, these different strategies facilitate the construction of specific identities *via* storytelling under divergent circumstances. But it must be kept in mind that some may have variations in our specific cases when narrators and audience do not remain in instant communications.

Furthermore, the study focuses on the primary proposition that translators’ recounting of their own working experience is a form of narrative. More specifically, it is a type of narrative knowledge. While narrating, translators as storytellers would make sense of their life by exploiting any resources at their disposal, including events, characters, places, and times, to name just a few essential narrative components.

## 3. Data of the study

The study selects five self-narratives primarily according to the high reputation enjoyed by their narrators in contemporary China’s literary translation field. In addition, other factors of gender, age, translation direction, and genres selected for translation are considered too. All five translators are born in the first three decades of the twentieth-century. Zhang Yousong (1903–1995) is best known for his translations of most of Mark Twain’s works, including *The Adventures of Tom Sawyer* and *The 1,000,000 Pound Bank-Note*. Xiao Qian (1910–1999) is remembered as a translator of *Tales from Shakespeare, The Good Soldier Schweik*, Ibsen’s *Peer Gynt*, and, more importantly, as co-translator of Joyce’s *Ulysses*. Zhao Luorui (1912–1998) won her fame due to the translation of American poetry, especially *The Waste Land* by T.S. Eliot in the 1930s. Yang Xianyi (1915–2009) has been primarily acclaimed as a senior figure in translating classical and modern Chinese literature into English since the 1940s when he returned to China with his wife Gladys Yang after a few years of study in Britain. Yang Jingyuan (1923–2015) won fame for her co-translation of *Karl Marx und Friedrich Engels: Leben und Werk* by Auguste Cornu. But the translation of children’s literature is what she treasures most in her narrative due to the reasons explicated in the next section.

Born in China during the first three decades of the twentieth-century, the five translators have spent their lives through the tumultuous periods of modern China. Their death rules out the possibility of conducting the interview for accumulating more data to support our interpretation. However, our analysis throughout will be primarily based on close and cross-readings of the narratives and the specific social–political–cultural contexts in which these narratives are generated. The five selections are provided in Table 1.

**TABLE 1:** Five translators’ narratives and publication information.

Numbers	Translators and titles of their narratives	Years of writing	Source collections	Editor(s)	Publishers	Years of publication
1	Zhang Yousong: random thoughts on literary translation (文学翻译漫谈)	1982	*One hundred translators’ talks on contemporary literary translation* (当代文学翻译百家谈)	Wang Shoulan	Beijing: Peking University Press	1989
2	Zhao Luorui: how do i translate literary works (我怎样译文学作品的)	1982				
3	Xiao Qian: treason, opening up and creation: translator’s preface on Chinese version of *Ulysses* (叛逆•开拓•创新—序《尤利西斯》中译本)	1994	*Ingenuity out of difficulties: famous translators’ talks on their translation experience* (因难见巧：名家翻译经验谈)	Jin Shenghua and Huang Guobin	Beijing: China Translation and Publishing Corporation	1998
4	Yang Xianyi: on translating of *The dream of the red mansion* (谈红楼梦的翻译)	1998				
5	Yang Jingyuan: memory of a unique “collaborative translation” experience (别具一格的“合译”)	2005	*One book and one world: translators’ written words on how world famous literary works “reached China”* (一本书和一个世界：翻译家笔谈世界文学名著“到中国”)	Zheng Lu’nan	Beijing: Kun Lun Press	2008

These narratives are from three Chinese collections, whose editors have claimed to put senior translators’ knowledge into anthologies so that “numerous professional translators, researchers, and amateur of foreign literature can use them as a reference” (Wang, 1989, p. 857). Moreover, readers will have a chance to appreciate “the talents demonstrated by great translators in transferring the original texts across language boundaries, and the prominent styles of their translations” (Zheng, 2008, p. 254). Thus, these collections primarily frame the archives as evidence of technique learning. The talks aim to boost the perception of the translation process, methods, and norms. Such an inclusive vision partially structures how the collected texts should be interpreted among targeted readerships. As expected, all the different narratives would be viewed primarily as how-to knowledge on translation; however, it is complex that the translation knowledge is narrated through life experiences and stories full of characters and events deemed significant to the storytellers.

Examination of the five narratives demonstrates that at the time of their completion, the storytellers are nearly in their 70s and 80s. Born in the first three decades of the twentieth-century, they have shared a similar experience in living through a restless China, which witnessed the revolutionary 1930s, the civil war between the Communist Party and Nationalist Party in the 1940s, the tremendous transformation initiated nationwide since 1949 when a new socialist China was established in congruent with Marxism, Leninism, and Maoism, and the tumultuous years at the height of Maoism before 1978 when China reopened its door to the outside world. Since the first several years of new China, the whole society has been increasingly structured into a class society where intellectuals have been officially designated as a group “mostly derived from the classes of landlords or bourgeois” (Zhou, 1951/2011, p. 442). Either in the emotion they express or in the language they speak and write, they have not integrated fully into the masses. “Thought reform” among intellectuals becomes a state policy (Yu, 2001; Chen, 2013).

As a result, they must receive re-education and transform themselves into the classes of laborers, peasants, and soldiers and serve the revolutionary causes of the Party, a mission clearly elaborated by Mao (1942) in his *Talks at Yan’an Conference on Literature and Art*. As intellectuals, the five translators in our cases have experienced similar adversity and tribulations in their life, especially during the years when some of their utterances in public media were criticized seriously in the Anti-Rightist Movement in 1957 and the 10 years of the Cultural Revolution from 1966 to 1976. The historical junctures of 1949, 1957, the Cultural Revolution (1966), and 1978 are frequently mentioned throughout our selected narratives, although mostly in an implicit manner. For example, Yang Xianyi was jailed for 4 years during the Cultural Revolution, while Zhang Yousong was also wronged as a Rightist in Anti-Rightist Movement launched in 1957. Hence, their life and work were utterly affected by the events breaking out one after another in the 30 years since 1949. Their narratives reflect their emotions, hopes, and sorrows in a complicated vein that needs to be examined with an intertextual reading across divergent contexts.

## 4. Analysis procedure

### 4.1. Narrative structures

The study uses qualitative analysis to analyze the data. Table 2 illustrates the scheme of components to be analyzed structurally. Repeated close readings of the five narratives reveal the thematic focus of each narrative. It requires first to “read the material several times until a pattern emerges, usually in the form of foci of the entire story” (Lieblich et al., 1998, p. 62). However, such qualitative and holistic reading can only work to its fullness by being integrated with the empirical method of breaking down a whole text into its components of smaller units, like the paragraph we adopted in this study.

**TABLE 2:** NARRATIVE analysis scheme.

Paragraph	NARRATIVE								
	Narrative								
	Characters	Time	Place	Activities	Events	Evaluative	Narrative stance	Analytic	Thematic

Table 2 indicates that NARRATIVE comprises small-lettered narrative, analytic, and thematic. Para stands for paragraph, arranged in order of how a narrative text is composed in its written form. Characters normally refer to animate actors in a story, like persons with whom the narrator has connectivity in his/her socialization. Sometimes non-animate actors like an institution and the Communist Party can also play a key role in translators’ narratives. Within this scheme, labels sometimes do not have absolutely clear-cut boundaries between each other. Overlapping is possible. For example, activities and events are closely related. The only difference is that activities are minor actions taken by characters. In contrast, events refer to a cluster of activities linked by some relations of cause and effect or logic. Evaluative indicates that storytellers often make a personal judgment of people, institutes, parties, actions, events, or social phenomena. It could be positive or negative.

The narrative stance leads us to consider what style a narrative is conveyed. Analytic indicates that the storytellers shift from storytelling to pure description of problems and empirical analysis of translation-related problems such as how to learn to be a qualified translator and what methods and strategies must be adopted in literary translation. Analytic barely involves events in specific temporal-spatial contexts so that a sense of objectivity is created. Hence, analytic contributes much to the scientific narrative. The last label thematic in our table is a summary of the main ideas in its corresponding paragraph. By dissecting all paragraphs into the schematic slots, each narrative will be presented as sampled in Table 3.

**TABLE 3:** First paragraph in five narratives.

Paragraph			1	1	1	1	1
NARRATIVE	Narrative	Narrator	Zhang Yousong	Zhao Luorui	Xiao Qian	Yang Xianyi	Yang Jingyuan
		Characters	I, comrades in translation circles, young readers with an aspiration for literary translation	I (我), my teacher (老师) Su Xuelin (苏梅苏雪林) my English teacher (英语老师)	I, translator Tian Han	I, Jin Shenghua 金圣华	I, my beloved husband, Kunlun Press, editor comrade, she
		Time	early twenties, post emancipation since 1949, now	at the age of 7, in one decade, at the age of 16 when entering university, in 1930 when second year in university ends	some day in 1942, in the late 1930s, in the days after the Attack on Pearl Harbor	in most time of this whole life	In the spring of 2005
		Place			Birmingham of England		
		Activities		My favorite teacher is Miss Su Xuelin who teaches me Chinese; I do not like English teacher.	In one day of 1942, I visited an exhibition of translated versions of Shakespeare.		
		Events	1 am invited to write a brief autobiography of myself.	In 1930 when I was in my second year at university I was persuaded by my English teacher to change my major from Chinese to English.	An exhibition exposes the backwardness in our country’s translation of classic works from foreign countries, thus exciting a strong sense of cultural patriotism within my heart.	Professor Jin Shenghua asked me to talk about my experience in translation.	I feel so much grieved by the passing away of my husband who was my company in 60 years that I am about not to live any longer. I am in no mood to write something about my translation experience, but the contact editor changed my mind with ner enthusiasm and sincerity.
		Evaluative	poor quality of my translation prior to 1949 when PRC is established; limited contribution; do not deserve to be called a famous translator; my opinion is only random conversation and shallow views for others to criticize and rectify; however, they may be of some value for reference.	The teacher who teaches Chinese is my favorite; I do not like English teacher; to study English is a burden to me because it brings me pressure from teacher ana distress.	Our country performs poorly in translating classic works from other countries, which illustrates our weak national power, and in particular our low level of cultural development and the populace’s cultivation. this is ‘very disgracing’!	My life-long engagement with translation enables me to talk about some experience, but it is lack of logic. I feel worried to talk about theory because I am not in a position to bring out deep insights. My opinion is translation is a kina of art or simply a technique. Of course this may be my biased understanding.	feel very much grieved; in no mood of writing
		Narrative stance	modest	factual	moral	moral	affected
	Analytic						
	Thematic		The narrator emphasizes the long time he spent in literary translation practice and thinks modestly that his experience may be of some value for others to consider.	Accidental shift to study of English prepares me for later work in translation.	I feel disgraced by our country’s weak cultural power with regard to translation of foreign classic works.	I would say something about translation, but my understanding is not much theoretical. It is merely empirical reflection on experience.	at the sincere request of the editor, I decided to write something about my stories concerning translation of two books.

Based on the structural methods of combining holistic-content analysis and the scheme illustrated in Table 3, the similarities and disparities between our five cases are identified. One is about professional quality flaunted or implied by translators themselves, either by enumerating how many and what important texts in foreign or Chinese languages they have translated or by mentioning how many foreign languages they have ever learned. The difference relies on which narrative stance they talk about it. Zhang Yousong displays modesty in his understated appraisal of the quality of his translations done before 1949 and in his reservation in acknowledging the usefulness of his experience-based talks to others.

By comparison, Zhao Luorui sounds more assertive and authoritative in advocating her literal translation method. She further argued that to those texts and writers that are not serious, “we should not go too far to treat them with excessive seriousness” (Zhao, 1982/1989, p. 608). Yang Jingyuan is emotional in tone primarily due to her husband’s death in his old age. The two translations she referred to in her narrative were direct products of their collaboration because she lost her eyesight caused by cataracts and had to rely on her husband to finish the translations. To her, the translations remind her of the happy days she and her husband worked together. Because of this event, her life becomes meaningful, especially in her later years.

The analogy across the five narratives is also identified in the way how the five narrators structure the chronological arrangement of the events happening in their life. Their stories are mostly deployed in three periods of time, that is, prior to 1949, the first 30 years since the foundation of socialist China, and the years from 1978 to the present time when their narratives were completed. Historical and political events like the Movement of Intellectuals’ Thought Reform in 1951–1952, the Anti-Rightist Movement in 1957, and the Cultural Revolution have exerted a great impact on their life and work. Narrators tend to employ the polarizing technique to draw a demarcation line between the abovementioned three periods so that things can be conceptualized as developing from bad to good, from negative to positive, and from adversity to bright prospect. Hence, a linear sense of progress surfaces. For example, Xiao Qian reflected in an affirmative tone that “it is hard to imagine having introduced Joyce into China in the thirty years since 1949” (Xiao, 1994/1998, p. 134) because of the incongruity between the poetics and ideology advocated by Joyce’s novel and those domineering in Maoist China. Yang Xianyi compares two important translating events divided by the year 1949. In 1943–1946, he and his English wife Gladys Yang worked at the Chongqing-based National Institute for Compilation and Translation as translators of Chinese classic and modern works into English. Since 1952, they were employed as professional translators in the Foreign Language Bureau, a Beijing-based institute responsible for introducing Chinese works of politics, society, literature, and art to the outside world. He asserted that the period from 1952 to the breaking out of the Cultural Revolution was witnessing his highest productivity in translating (Yang, 1998, p. 81). Zhang Yousong went further to ascribe the improved quality of his translation after 1949 partially to the leadership of the Communist Party.

While in the years after 1978, he was convinced that “with correct leadership of the Communist Party, any kind of work could be completed without taking a roundabout route” (Zhang, 1982/1989, p. 439). Apart from polarizing between different time periods, Zhang also attempts to create a binarism between senior translators of his generation and the youth translators. The purpose is, among others, to highlight senior translators’ rich experience and hence their value in training the latter for the enterprise of literary translation in China. In doing so, Zhang asserted the meaning of his work for the people and the Party, which brings him “ultimate happiness.”

The biggest difference between the narratives under discussion probably lies in how they arrange analytics. These analytics involve the translators’ perception of their work and how-to questions about translation. Thus understood, they are tantamount to pure translation knowledge, a body of concepts, prescriptions, and procedures on translation. It can be understood as a type of disciplinary narrative. Table 4 presents the percentage of paragraphs of analytics within the total number of paragraphs in each narrative. It is our hypothesis that the less percentage of the analytics in a narrative, the more personal the narrative tends to look as a whole. As generally viewed, Yang Jingyuan’s narrative among the five is most personalizing because the majority of her account involves her attachment to her husband.

**TABLE 4:** Percentage of analytics in each narrative.

Numbers	Storyteller	Total number of paragraphs (TNoP)	Total number of paragraphs of analytics (TNPoA)	TNPoA/TNoP (%)
1	Zhang Yousong	29	16	55.2
2	Zhao Luorui	19	13	68.4
3	Xiao Qian	124	32	25.8
4	Yang Xianyi	11	5	45.5
5	Yang Jingyuan	17	1	5.8

With a view to the five specific cases, this research itself can also be viewed as a narrative with its hypothesis of not claiming 100% scientific and objective study but striving for a balanced and nuanced articulation of translators as persons living in the specific sociopolitical environment. We tend to offer a complicated understanding of the world in which translators have inhabited. It is our conviction that the ultimate objective of knowledge production is to increase the goodness not merely for the collective, but also for individuals, for those persons who can feel and err in real life.

### 4.2. Identity construction

Identity is a discursive construction. Translators use whatever sources of language, characters, and events are at their disposal to create a meaningful ensemble for themselves and others. In our five selected cases, the beginning and end of a narrative contribute most to shaping the identities. While in the middle part of a narrative, what key events should be arranged can also lead to the successful construction of a translator living in specific social-and-political milieus.

A narrative’s beginning in Labov and Waletzky’s structural analysis of oral versions of personal narratives is reconfigured as orientation, which serves to “orient the listener in respect to person, place, time, and behavioral situation” (Labov and Waletzky’s, 1967, p. 32). The results of our analysis indicate that the beginning is an initiation because it functions to state the reasons why narrators start to engage with translation. Zhang Yousong set the beginning of his translation activity in the early 1920s. The second paragraph in his narrative presents more details about the incident that pushed him to get involved in translation. When he was young, his father died. The heavy pressure on his shoulder to take care of the whole family seems to play a pivotal role in his final decision to select English as his major. For the same reason, he tried his luck in translation before graduating from university to earn a living. In the period prior to 1949, he expressed a paradoxical attitude to his translation fulfillment.

On the one hand, he thought highly of his success in translation. He stated, “I have published a couple of translations before graduation from university, thus gaining some reputation in the translation circles at that time. From 1928 to 1930, the Chun Chao Publishing House I ran in Shanghai ended with a failure, but the translations I generated enjoyed a good market” (Zhang, 1982/1989, p. 431).

On the other hand, as implied, he tried to create a hierarchical self-evaluation of his work between two periods divided by 1949. However, in the foreground, his achievements and endurance against hardships and adversity would consolidate the impression of his tenacity and courage, especially in face of life’s vicissitudes throughout history. This inference is justified by the gratefulness he expressed for what he had been maltreated during the years at the height of extreme leftism afflicting China, especially in the latter part of the 1960s. He finally concluded that “my bitter experience, however, is not worst, because I have finally survived the miseries and luckily enough, I am now not disabled physically and can continue working” (Zhang, 1982/1989, p. 434). Except for the critical event in his early years, other events like his transfer to a post in People’s Literature Press as a professional literary translator and the historical event of China’s opening up and reform have also positively transformed his life and work. While the political movements during the 30 years since 1949 bring many unhappy memories to him. Even though he has had such unfair treatment due to political incorrectness, he remains devoted to working as a translator with a high sense of mission. His narrative constructs him as a man of integrity in terms of work ethic.

Zhao Luorui, as partially illustrated above, sounds more assertive in her professional competence as a translator. The key event in her life before 1949, and to a certain degree in her whole life, is the 4 years of study at the University of Chicago from 1944 to 1948. She claimed that “the 4 years at Chicago University have utterly changed my way of thinking and research, enabling me to completely transform to a method of perceiving matters from analytical, rational, and objective perspectives” (Zhao, 1982/1989, p. 606). After her graduation from Chicago University, she returned to China, and 1 year after, the PRC was established. In the early formative years of a socialist China, the Thought Reform Movement has been launched with the aim to help all intellectuals, especially those with an overseas education background in England and America to criticize their capitalist ideologies and transform themselves into Marxism, Leninism, and Maoism. Zhao’s 4-year study in America made her a target to be criticized in the early 1950s. This is the reason why she reiterates in her narrative that what she studied in the 4 years is not contradictory to Marxist and Leninist conceptions of literature and art. On the contrary, the two are complementary to each other because “Chicago teaches me the method and technique, while Marxist and Leninist ideology of literature and art give me a correct stance” (Zhao, 1982/1989). Both intellectual sources, she further argued, have exerted a positive influence on her conception of translation.

Xiao Qian’s narrative, as will be illustrated here, focuses on the original novel of *Ulysses* and the reasons why he made his final decision to translate it and the methods he and his wife selected in their translation. Unlike the rest four narratives, Xiao’s account is originally aiming at introducing the novel he translated to its potential readership, so he did not mention anything about his lifelong stories involving translation but began his stories about his translated novel from the 1940s when a critical event triggered his strong sense of cultural patriotism, which is partially contributing to his final decision to accept an invitation to engage with the translation of *Ulysses*. In addition, he reiterated the necessity of introducing this novel to China because he argued here and there in his account that its innovation in the adoption of “stream of consciousness” would benefit Chinese writers, even though the narrative technique employed in the novel is not congruent with his conceptualization of literature and art since he believed that art for life rather than art for art’s sake is what China needs most.

Throughout his narrative, his personal life experience is mentioned only when it has connectivity with the novel itself, for example, in his education from 1929 to 1930, he happened to know the name of James Joyce in the courses taught at universities in Beijing. Most stories in his account are about Joyce and his literary creations. Analytics concern with his explanation about the preparation and translation of the novel by him and his wife. He unlike other translators did not mention how many translations he has produced as proof of his professional competence. However, such expertise by a translator is illustrated by the description of how much he and his wife have devoted to solving many difficulties encountered in their translation process, such as issues of multilanguages used in the original, divergent dialects, background knowledge involved, and if notes should be added to make the novel’s content more explicit to Chinese readers.

A few incidents deserve our attention since they contribute most to how we understand Xiao’s identity. First, he seems to stress the fact that the novel itself is very abstruse to translate due to its special narrative technique, innovative use of language, and rich cultural background knowledge involved. He concluded that it is fortunate to complete the translation. The whole process of translation is like “attacking and occupying a fortress.” The emphasis on how difficult it is to translate the novel indirectly foregrounds Xiao as a professional translator with enough capacity to do pioneering work. The work Xiao and his wife have devoted to translating this novel also indicates the high work ethic expected to be possessed by translators due to the mounting difficulties imposed by this novel. Second, Xiao views the completion of this translation as a collaboration with his wife and many more people who offer their assistance. On the one hand, he accepts that it is not possible to have this work translated into Chinese in the years from 1949 to 1978 due to the height of Maoism, which values a work like this in a negative vein. In contrast, its successful translation signals the degree to which China takes an open attitude toward literature and art derived from capitalist countries and cultures. More importantly, Xiao views this translation as a scenario that witnesses how with his wife’s assistance, he has learned “to transform myself from an indolent person to a diligent man. Translation of *Ulysses* is the summit of such transformation” (Xiao, 1994/1998, p. 132–133). In different periods from 1949 to 1978, Xiao suffered from political persecution particularly in the Anti-Rightist Movement in 1957 and 1958, and the Cultural Revolution. However, this experience is not mentioned directly in his narrative here, except that a couple of concepts like “corrective remaking” (改造) will remind us of the political movements rising in the tempestuous moments in China since the 1950s.

The implicitness in commenting on past events gives us a chance to think deeply about the meaning between the lines. As Xiao comments: “In about seventy years, there is only one novelist called Joyce known in the West. This example demonstrates that what gets affirmed and praised is not necessarily meant to be the path and direction for all others to follow. Artistic creation can only be the result of personal wisdom and the mirrored image of people’s spirit” (Xiao, 1994/1998, p. 135). Put against the Maoist conceptualization of literature and art for the classes of laborers, peasants, and soldiers, Xiao’s comment can be understood as an implicit critique of the popular conceptualization and practice of treating literature and art as a tool for political sake. In sum, Xiao looks like a devoted translator with a strong sense of cultural patriotism and a persevering support of literature and art for life’s sake. His assertion of literary translation for Chinese literary creation constructs an identity of a realist artist devoted to the cause of enriching Chinese literature and art.

Yang Xianyi’s narrative has a clear chronological line of development, starting from his middle-schooling years to the time when his narrative was written. His life can be conceptualized into three main periods: prior to 1949, from 1949 to the 1980s, and the recent several years of the 1990s. In his description of his first period, he tends to highlight his talents in studying different foreign languages, including English, Latin, Greek, Spanish, French, and Sweden. In addition, his early attempts at random translating and the 6 years he spent studying in England are also mentioned as proof of the capacity a translator is supposed to possess. However, in this period, the most important experience for him is when he and his English wife Gladys Yang worked together as translators in the 1940s for the National Institute for Compilation and Translation established in 1932 by the Education Ministry of the National Government. In the 3 years of working in this institute, he and his wife translated a great number of Chinese literature into English. The second period, especially from the 1950s to the breakout of the Cultural Revolution in 1966, is the time witnessing his richest production of translations of Chinese literature into English. In the analytics, he elaborates on a couple of issues relevant to translation, including translatability, cultural differences, translation principles, and poetic structures and rhythms. The most interesting part is his reference to an incident about his encounter with Chairman Mao in a meeting where Mao asked him whether Qu Yuan’s *Encountering Sorrow* (离骚) can be translated across different languages. He blurted out yes and replied that “no texts are untranslatable.” The citation of this incident in his narrative is important because the connectivity Yang has with the then top leader of China may serve as proof of his authority as a translator. In terms of the length of the narrative, Yang’s is the shortest, the reason for which may be due to his weariness of repeating his perception of translation. He even wished that “this could be the last time for me to talk about translation” (Yang, 1998, p. 84). This reservation may be also caused by the self-evaluation of his lack of theoretical thinking even with rich empirical experience.

Finally, Yang Jingyuan’s narrative centers on two translated works *The Brontë Stories* and *Peter Pan* (Yang, 2005). The latter has a connection with her life prior to 1949, while the former has much to do with the stories happening during her long years of suffering from cataracts and how her husband accompanied her to complete the translation of this book. As usual, her life consists of years prior to 1949, the period from 1949 to 1978, and the days after 1978 when China adopted a national policy of opening up and reforming. In the third period, the year 1984 is unique because she was afflicted by a cataract and, thus, lost most of her eyesight. This illness deprived her of devotion to translation and research. However, her husband recommended himself in helping her to continue her translation. With his help, Yang Jingyuan began a special co-translation of *The Brontë Stories*. Her husband reads the original text sentence by sentence. She is responsible for orally translating them into Chinese, and then her husband will write them down for later proofreading and mutual revision. In doing so, they spent happy days together in the parks, hotels, and trains on their journey to other cities or while they stayed at home. This special collaboration in translation proved a success since the translation has received positive feedback from readers. Then they continued to do the translation of her favorite children’s literary text *Peter Pan*. Yang Jingyuan did not mention too much of her expertise as a translator in her narrative, such as how many texts she translated and what qualities she had for engaging in translation.

It is noticeable that she expressed the contradiction between her dream of becoming a novelist and the reality of taking translation as her primary career. In the second period, what she worked for is the translation of social sciences and politics which she later admitted are not her interest as an incident indicates that she was someday assigned to join a translation team with other two members to translate Marx and Engles’ biographies from English into Chinese. Though this translation activity occupies an essential role in her work as a translator, she expressed her reluctance to do it because her favorite was literary translation. By drawing polarized boundaries between likes and dislikes, she assigned positive meaning to the life experience with her beloved one. In sum, translation becomes meaningful in her later years when it brings her sweet memory of happy togetherness with her most intimate family member.

It happens that how a narrative ends itself can, in one way or another, influence how we interpret the stories as a whole. Labov and Waletzky referred to Coda as “a functional device for returning the verbal perspective to the present moment” (Labov and Waletzky’s, 1967, p. 39). But the five cases of this study demonstrate that narrators differ significantly in which directions they move the endings of their narratives.

As Table 5 conveys, the narratives in their endings return their perspectives to the past, the present, and the future. The three perspectives would generate different meanings. Backward looking adds strands of emotions to the whole story because memories of past events can help narrators to make meaning of their present existence. Similar to Yang Jingyuan’s ending, her life’s meaning would more likely lie in the two collaborative translations that refer to the past when her husband was with her. Translation becomes meaningful merely due to its connectivity with her beloved mate. But in Xiao Qian’s ending, the nuance is that translation *per se* becomes important rather than the narrator’s relative, his Third Sister. Thus, Xiao’s narrative focuses on stories about the novel, the original writer, and the translation process. Moreover, a forward-looking vision typically contributes to constructing a strong sense of optimism and a myth of linear progress. However, Yang Xianyi’s sense of the future differs greatly from that of Zhang Yousong’s in that the former shows much reservation about continuing his narration of translation experience, while the latter prepares to work at his full capacity for the meta-narratives such as the national mission and interests for the people and the Party. Comparatively speaking, Yang Xianyi tends to be more detached from translation while Zhang Yousong appears more engaged in related activities. Zhao Luorui’s near-future angle enables her a high possibility of spreading her ideas on translation if another chance is provided.

**TABLE 5:** Different endings of narratives.

Narrative	Narrator	Ending	Type
1	Zhang Yousong	Our publishing units should give those easy works to our young translators, so they may have opportunities to have a practice. Those magazines specializing in foreign language studies can also publish some short passages of foreign languages for young people to translate. Hence the best can be selected for publication. In a word, all sides involved should find more ways to train new forces. If only all present forces can be mobilized to make the literary translation community grow *via* combining the different groups of senior, middle-aged and young translators, our mission of literary translation is expected to have bright prospect (p. 440).	Future
2	Zhao Luorui	My translation of *Leaves of Grass* by Walt Whitman has just commenced. I am hoping that in the future I have chance to make a summary of my little experience as a translator of literary works (p. 614).	Near present-near future
3	Xiao Qian	To conclude, we have one more person to express our gratitude. She is our third sister who passed away today in last year. This translation is dedicated to her because without her strong support, we could never afford to accept such a heavy task of translation (p. 137).	Past-backward looking
4	Yang Xianyi	I have recently busied myself with moving into a new apartment. At the request of Professor Jin Shenghua, I scribbled a few thousand words. I should feel ashamed of talking about ideas of commonplace. I am now over 80 years old. It is high time for me to stop writing anything more. I do hope that this is the last time for me to have a talk about issues on translation (p. 84).	Near present-future
5	Yang Jingyuan	At the end of 2004, “*Peter Pan*” this happy boy who are always a kid spent his 100 years birthday. However, my husband of life-long company and I have been afflicted by illness due to getting old. In early 2005, my old mate finally let go his hold and left me. Sixty years of helping each other in distress and 60 years of tasting sorrow and joy are all gone like a puff of smoke barely leaving any trace at all. What I have in my hand is just the two published translations which were completed with him to read the original texts sentence by sentence and write down the whole texts word by word. They are memorials that will last forever (p. 16).	Past-backward looking

## 5. Conclusion

The study concludes first and foremost that translators as a group has shared some similarities in their identities. They tend to personalize their experience to foreground the qualities required of translators. In addition, it reveals that professionalism is key to the conceptualization of translators’ identities. Translators would resort to whatever possible resources to justify their professional competence. Key events are recalled. A wide range of texts they translated is enumerated. However, it needs to be clarified that professionalism involves more than linguistic competence. Translators would polarize divergent resources like a chronological sequence of events, hierarchical structures of social groups, or relations between environments and people so as to construct themselves as persons with high integrity, best illustrated by their allegiance to work ethics as translators.

The study further implies that specific social–political–cultural settings where translators have lived would have a deep imprint upon individuals’ life and work and play a pivotal role in shaping what meanings they could make of their sufferings, adversities, sadness, and happiness. All translators in our study have had to subject themselves to a series of significant historical events breaking out in modern China, especially at critical conjunctures in history. The year 1949 witnessed the establishment of socialist China as an independent country. It has finally chosen to join the alliance led by the former Soviet Union. Due to the restrictive ideology of Marxism, Leninism, and Maoism, the Chinese government soon launched various movements to transform intellectuals in line with the Maoist reconfiguration of literature and art for social, political, and cultural considerations. Individuals’ fate is so closely tied with the nation’s that personal narratives get twined with public, conceptual, and meta-narratives. Translators personalize their stories for the public presentation of who they are or to answer a missionary call from meta-narratives popular within given environments.

In identity construction, unity nurtures diversity, presenting before us a colorful spectrum featured with Zhang Yousong’s Prometheus spirit of incessant working, Zhao Luorui’s rational and authoritative position, Xiao Qian’s assertion of cultural patriotism and literary realism, Yang Xianyi’s detachedness, and Yang Jingyuan’s affective attachment. By employing divergent narrative functions and strategies, narrators maneuver time and events to tell multifaceted stories about themselves and collective interests. We would like to conclude that the narrative approach to translation could extend its current focus on conflictual and synchronic respects to everyday activities and diachronic dimensions informing how translators make meaning of their life and work *via* storytelling. The shift to human-centeredness exemplified in this article will serve as an alternative vision with which to gain a complex perception of what translation could possibly mean to its practitioners. More importantly, translators’ self-narratives are no longer viewed merely as a disciplinary narrative about translation knowledge. They involve more than the conceptual or analytic accounts of translation issues. In fact, they function to instruct, to educate, to entertain, to memorize, and even to forget.

## Data availability statement

The raw data supporting the conclusions of this article will be made available by the authors. Requests to access the data should be directed to QG, gengqiang@shisu.edu.cn.

## Author contributions

The author confirms being the sole contributor of this work and has approved it for publication.
